# Improvement of female pattern hair loss with bicalutamide: A time-dependent process

**DOI:** 10.1016/j.jdin.2025.07.008

**Published:** 2025-08-29

**Authors:** Rocío Gil-Redondo, David Saceda-Corralo, Juan Jiménez-Cahué, Sergio Vañó-Galván

**Affiliations:** a Hair Disorders Unit, Grupo Pedro Jaén, Madrid, Spain; b Department of Dermatology, Hospital Universitario La Paz, Madrid, Spain; c Department of Dermatology, Hospital Universitario Ramón y Cajal, Madrid, Spain; d Departamento de Medicina, Facultad de Medicina, Universidad de Alcalá, IRYCIS, Madrid, Spain

**Keywords:** bicalutamide, female androgenetic alopecia, female pattern hair loss, hypertrichosis, minoxidil

*To the Editor:* We read with interest the study by da Silva et al, which evaluated bicalutamide 25 mg/day plus minoxidil 1 mg/day versus minoxidil alone for female pattern hair loss (FPHL). While no improvement in hair density was observed with the addition of bicalutamide, the study did report reduced hair shedding and facial hypertrichosis.^1^ We believe several aspects warrant further discussion to better contextualize these findings.

Bicalutamide is a selective androgen receptor (AR) antagonist with a more favorable safety profile than flutamide and a distinct mechanism of action compared to 5-alpha-reductase inhibitors. Unlike spironolactone, it lacks mineralocorticoid activity, making it a potential alternative when other antiandrogens are poorly tolerated (Table I).^2^

**Table I:** Comparison of antiandrogenic drugs for the treatment of female pattern hair loss

Characteristics	Bicalutamide	Spironolactone	Finasteride	Dutasteride
Mechanism of action	Selective androgen receptor antagonist	Aldosterone antagonist with antiandrogenic activity (inhibits androgen production and receptor binding)	5α-reductase type II inhibitor (reduces DHT production)	5α-reductase type I and II inhibitor (more potent than finasteride)
Most common side effects	Liver enzyme elevation, fatigue, hot flashes	Menstrual irregularities, breast tenderness, hyperkalemia	Decreased libido, headache, breast tenderness	Same as finasteride, may include higher rates of libido reduction
Efficacy data in FPHL	Retrospective studies show Sinclair stage improvement up to 28% at 24 months	Studies report improvement in Sinclair scores in 40-60% of women after 6-12 months	Modest improvement in some studies, variable efficacy in premenopausal women	Higher efficacy than finasteride in some reports
Discontinuation before pregnancy	3 months	1 month	1 month	6 months

*FPHL*, Female pattern hair loss.

The study’s main limitation is its short follow-up period (24 weeks), which may be insufficient to detect meaningful changes in hair density in FPHL. Antiandrogen treatments often require longer durations to show clinical efficacy, as supported by prior research.^2^ The reduction in hair shedding observed in the bicalutamide group may indicate early biological changes such as anagen phase prolongation and miniaturization reversal—events that typically precede visible density improvements and would be better captured with longer follow-up.

In the largest retrospective study on bicalutamide (138 women), progressive improvement in Sinclair stage was noted over 2 years: from 0.18 at 3 months to 0.80 at 24 months.^2^ These data support assessing antiandrogen therapy outcomes at 1-2 years rather than within a few months.

The small sample size in the da Silva et al study (32 participants per group) may also limit its statistical power to detect differences in hair density. Future randomized controlled trials should incorporate predefined power analyses, larger samples, and longer treatment durations. Real-world studies and meta-analyses could also enhance understanding of long-term efficacy and safety.

Of note, bicalutamide appeared to prevent minoxidil-induced facial hypertrichosis. This effect supports its antiandrogenic activity and aligns with prior findings on bicalutamide.^1^ Facial hair may respond more quickly to antiandrogens due to biological differences between facial and scalp follicles. These include shorter anagen phases in facial hair (∼3 months vs 2-8 years in scalp hair), higher AR expression, and regional variation in 5-alpha-reductase activity and isoform predominance.^3^^,^^4^ Additionally, facial follicles express more AR coactivators (eg, ARA70), which enhance local androgen signaling. Epigenetic factors also play a role: scalp areas less affected by FPHL show greater AR gene promoter methylation, potentially reducing AR expression and conferring resistance to androgens.^5^ These factors likely contribute to the slower and often incomplete response of scalp hair compared to the more rapid response in facial hypertrichosis.

In conclusion, while da Silva et al's study provides early evidence of bicalutamide’s clinical effects, the short follow-up limits conclusions about efficacy in hair density. We support considering bicalutamide in select FPHL cases, especially where other antiandrogens are not suitable, while encouraging longer-term trials.

## Conflicts of interest

Dr Gil-Redondo declares receiving honoraria from Eli Lilly, UCB, and Cantabria Labs as a speaker. Dr Jiménez-Cahué declares receiving honoraria from Eli Lilly, Amgen, Almirall, and Cantabria Labs as a speaker. Dr Saceda-Corralo declares receiving honoraria from Pfizer, Eli Lilly, and Cantabria Labs as a speaker and advisor. Dr Vañó-Galván declares receiving honoraria from Pfizer, Eli Lilly, Pierre Fabre, and Cantabria Labs as a speaker and advisor. All conflicts of interest are unrelated to the submitted work.
